# Whole of Community Facilitators: An Exemplar for Supporting Rural Health Workforce Recruitment through Students’ Professional Experience Placements

**DOI:** 10.3390/ijerph18147675

**Published:** 2021-07-19

**Authors:** Sandra Coe, Annette Marlow, Carey Mather

**Affiliations:** 1 College of Health and Medicine, University of Tasmania, Locked Bag 1322, Launceston, TAS 7250, Australia; sandra.coe@utas.edu.au; 2 School of Nursing, College of Health and Medicine, University of Tasmania, Locked Bag 1322, Launceston, TAS 7250, Australia

**Keywords:** rural, health, workforce, nursing, education, placement, supervision, clinical, community, facilitation

## Abstract

The Whole of Community Facilitator model provides support for healthcare students’ professional experience placements (PEP) in rural regions in Tasmania. In Tasmania, rural PEP is challenged as healthcare facilities are often small and have limited capacity for staff to devote considerable time to supervising students during PEP. Recruitment and retention of the rural health workforce in Tasmania is sometimes difficult because the island State is geographically distant from mainland Australia, and predominantly classified as a regional, rural, or remote area. The University of Tasmania, College of Health and Medicine (the College) explored various initiatives to support rural workforce sustainability, and the project discussed addresses this issue by promoting rural healthcare facilities as potential employment destinations for students upon completion of their course. The model supports the delivery of high-quality supervision to students whilst undertaking rural PEP, to foster positive experiences and potentially influence their future career choices. A successful exemplar was trialled in 2012 and implemented statewide in 2017 using a Whole of Community Facilitation (WOCF) model. The initiative supports host facilities, supervisors, host staff, and students and promotes positive placement experiences. The initiative was designed in consideration of Tasmania’s rurality, and uses a flexible and responsive framework.

## 1. Introduction

The Whole of Community Facilitator (WOCF) initiative arose from a novel experimental model of PEP facilitation trialled in 2012 [1], and further developed into a statewide initiative that takes a whole of community perspective to support healthcare students and their hosts during rural placements. PEP is the acronym used to refer to “professional experience placement” that defines a period of practical experience in a real-world setting. The underlying objective of the WOCF initiative is to increase the future rural healthcare workforce. The project is novel because it uses a broad approach with support directed at host staff and supervisors as the primary means through which students’ placement experiences are improved, including the quality of supervision received. In this way, the initiative aims to indirectly influence students’ future career choices and encourage their return to placement communities after graduation, and thereby achieve the primary aim of increasing the potential future rural healthcare workforce [2]. The sustainability of rural healthcare workforces is an issue of significant importance, and the Australian Government has invested heavily in initiatives attempting to address this concern.

Attracting and retaining an appropriately skilled workforce, along with the impacts of an ageing population, are challenges facing the rural health sector in Australia [3]. From the early 1990s, the Australian Government has funded a variety of programs attempting to address these issues of rural health service provision [4]. The Rural Health Multidisciplinary Training Program (RHMTP): Dentistry, Nursing and Allied Health—Rural Student Placement Expansion Project is an Australian Government initiative that provides funding for activities that expand and support rural placement experiences for domestic undergraduate students in healthcare disciplines to increase students’ exposure to rural healthcare settings [5]. RHMTP funding supports the initiative presented in this paper, including the employment of the Whole of Community Facilitators (WOCFs) central to the initiative.

Australia is not unique regarding the challenges to rural health services. Research suggests that, internationally, rural health services struggle to maintain sustainable rural healthcare workforces [6,7,8]. Jones et al. (2019) estimate there is currently a shortage of an estimated 10.3 million workers from the global healthcare workforce, with at least 7 million of these from the rural healthcare sector [7]. Nurses represent the largest proportion of health professionals within rural healthcare workforces [7]. Hence, the WOCF initiative provides a useful exemplar for improving the quality of students’ placement experiences and potential future rural nursing workforces, both nationally and internationally.

The challenges of rural workforce sustainability are of particular importance for the Australian island State of Tasmania. The State has the third lowest population of all Australian States and Territories (estimated to be 528,201 in 2021) and encompasses the smallest geographical area of 68,401 km^2^ [9]. Using the Australian Standard Geographical Classification—Remoteness Area (ASGR-RA), 40% of Tasmania’s population live in regional, rural, or remote areas [5]. Therefore, there is a large demand on the rural health sector with a low and decreasing pool of potential future healthcare workers. This places considerable strain on current health services and service providers [10].

Research indicates a positive rural placement experience as a student can strongly influence their graduate career choices, both in Australia and internationally [8,11,12]. Graves (2018) termed this influence “a dose–response relationship”, suggesting that a student’s length of exposure to rural practice settings correlated with future career choices regarding rural practice [12]. Paying attention to the quality of student placements, and increasing the number of healthcare students allocated to rural placements, must be important considerations in a broader global strategy for addressing workforce shortages in rural communities [10]. Given this imperative, research indicates that a significant influence on students’ placement experience is the supervision they receive during placements [13].

### 1.1. Professional Experience Placement

In Australia, nursing students must complete a prescribed number of hours of practice-based work-integrated learning, known as professional experience placement (PEP), to meet the requirements of their course of study. Nursing students undertake multiple PEPs throughout their degree and are placed in a variety of healthcare settings to ensure a depth and breadth of experience. Potential graduate nurses seeking registration need to complete a minimum of 800 h of PEP as students within their course, and that experience must be supervised to ensure safety and protection of the public, and to provide mentorship, guidance and direction [14].

Professional experience placements immerse students within real-world healthcare environments, and there are multiple variables that affect each PEP, making every experience unique. Some of these variables include the leadership, management, and work-place culture or availability of resources at a host facility [15,16]; type of healthcare facility and recipients of care; personal factors and expectations of students and staff; time of year; weather conditions, and geographical location [17]. In addition, particularly with rural PEP, students can often be supervised by different staff members each shift [18]. Furthermore, often there is only one student scheduled to attend a small rural healthcare environment, whereas other settings may host multiple nursing students, or students from other health professions at one time. Although not an exhaustive list, these variables influence students’ understandings of rural healthcare environments, resulting in unique experiences for each student.

Quality supervision is crucial to positive placement experiences as it fosters supportive learning environments, enables acquisition and development of clinical skills, and provides a psychologically and physically safe environment for students to practice [19]. Two well-known supervision models used to support nursing students in healthcare settings in Australia are preceptorship and clinical facilitation [14,19]. Within nursing, “preceptorship” describes the supervision of students in healthcare settings by registered nurses. Preceptorship occurs within the context of preceptors’ usual work environment with the student practising alongside, and being guided by the preceptor as they attend to their everyday nursing practice. This supervision usually occurs on a 1:1 or 1:2 basis, and preceptors act as role models and guides to students [20,21]. This model can be labour-intensive and increases the work and emotional demands for staff in supervisory roles, making it problematic for already understaffed rural facilities [1,6,21]. Moreover, staff in rural facilities are often part-time or casual, and students can have different preceptors or supervisors with each shift. This can affect the quality of the student’s supervision and their overall placement experience [1].

Australian research suggests that nursing students learn best with the clinical facilitator (CF) model of supervision [22,23]. CFs oversee student placements and undertake supervision, evaluation, and assessment activities [24]. CFs’ priorities are student-focused [17,24] and they may work directly with students, although students primarily work alongside, and are supervised by, registered nurses within host facilities. Unlike preceptors, CFs can oversee the learning and teaching of up to eight students during a single placement [24]. To facilitate positive PEPs, CFs provide learning interventions, observe, support, debrief, and give feedback to students [22]. CFs also inform healthcare staff about students’ learning needs, and liaise with the education provider, host organisation, and students during PEP [22]. For these reasons, the CF model was used for the initiative as it presents the most promise as a framework to positively support students’ rural PEP.

As supervision is necessary for PEP, the quality of this activity is of primary importance, particularly for rural placements, to positively influence students’ future career choices. Education providers strive to ensure students experience quality placements, although they often have little control over the quality of that supervision during PEP [6]. To guide quality supervision during PEP, continual and consistent support of supervisors is essential [25]. Hence, the CF model was chosen as the most appropriate model for the initiative, so that support would be afforded where it could provide the greater impact—through the supervisors and staff working directly with students on placements.

### 1.2. Determining Quality Supervision

As the quality of supervision during PEP can significantly influence students’ future career choices and, by default, the potential future rural healthcare workforce, there is sustained interest in developing contemporary PEP supervision models that encourage quality supervision [14]. Cognizant of this notion, a range of tools have been developed to measure quality. In Finland and the United Kingdom, Saarikoski et al. (2008) have developed the CLES (Clinical Learning Experience Scale) [15], which has been adapted into the CLES T Scale for use in Austria [26]. The Placement Evaluation Tool (PET) [27], and the Quality Clinical Placement Evaluation (QCPE) tool [28] have both been developed in Australia.

Alternatively, the term “best practice” is used as a quality indicator with a range of frameworks developed to improve the overall quality of PEPs. In Australia, the Best Practice Clinical Learning Environments (BPCLE) framework, and its revised version, the Clinical Learning Environments Evaluation Framework (CLEEF), have been widely adopted by state departments and national organisations [29]. The BPCLE framework has also been strongly recommended by Siggins Miller Consulting (2012) following its robust review of the elements of quality clinical placements [19]. Perry, Henderson, and Grealish (2018) also undertook a systematic review of supervision best practices to explore the behaviours of nurse supervisors who facilitated and promoted student accountability for learning during placements [30].

The key elements identified across national and international literature on quality and best practices in placement supervision can be summarized into two categories: enablers and barriers. Enablers to positive supervision experiences include:

- Capability of supervisor—connected to knowledge and experience [28].
- Workplace culture (or “atmosphere”, as referred to in international literature) of host organisation includes, values learning, welcoming and friendly environment, and management/leadership style [15,28,30].
- Students being prepared for placement prior to PEP, including attitude towards placement site, and orientation [31].
- Respectful relationship between supervisor/s and student, including communication and collaboration [15,20,30].
- Learning opportunities linked to direct patient care, includes incremental clinical independence and allocation of tasks to capabilities of student/s [20,30].
- Access to appropriate resources [30].
- Consistency includes supervision and a primary contact/support person for student/s [20].

The issues identified as barriers to positive placement supervision and experiences are:

- Supervisor’s normal clinical workload impeding supervision capability [18].
- Part-time nature of supervisor’s employment [18].
- Staffing shortages [18].
- Workplace incivility, aggression, and/or exclusion and gossip [30].
- Discrediting students as “learners”, including assigning menial and repetitive tasks or tasks beyond student’s capabilities [30].

With these enablers and barriers considered, the WOCF model was developed to provide support in a way that accounts for the community of PEP stakeholders, including host organisations, managers, supervisors, and other staff, as well as students, and the educational institution. The enablers and barriers are also used as benchmarks to reflect on the quality of supervision during PEP, as discussed in the research feedback.

## 2. The Research

Acknowledging the enablers and barriers to successful student placement experiences and to promote best practice in supervision of students in rural areas, the Whole of Community Facilitator (WOCF) initiative arose from a range of programs exploring alternative forms of support for host supervisors and nursing students during PEP [1]. Given the lower numbers of students placed in rural areas across Tasmania, replicating the model of supervision afforded to students in larger metropolitan areas was often not possible. Therefore, in 2012, an innovative model was trialled across two study areas in rural Tasmania. This early model led to the development of the WOCF model, which was initiated statewide in 2017. The program employs up to 13 WOCFs who provide support to host facilities, supervisors, and students before, during, and after PEP. Since its implementation in 2017, the program has evolved and matured to become an embedded and vital feature of PEP support in rural Tasmania.

### The Methods

Using action research and an iterative process [32,33] from 2012 to 2020, the initiative was evaluated via mixed methods using qualitative and quantitative data analyses of interviews and online surveys of managers, supervisors, other staff, and students. Ethics approvals for the research activities were obtained from the Human Research Ethics Committee (Tasmania) Network (H0016227 and H0018166) [1]. The 2012 trial, previously published [1], demonstrated how a CF assigned to an entire region with multiple placement sites could be instrumental in affording students a wholistic understanding of a geographical area, rather than a single location. It also opened pathways of possibility for small, non-traditional facilities to host students during PEP. Although many of these healthcare facilities did not become regular PEP hosts, some facilities did [1].

Alongside the 2017 implementation of the initiative, staff at host facilities, the newly appointed WOCFs, and students enrolled in the nursing program were invited to participate in individual or group interviews. Recruitment occurred via email and referral, as WOCFs made themselves and the initiative known to the healthcare facilities in their region. The purpose of the 2017 interviews was to explore respondents’ experiences of PEP. It was anticipated the results would provide a reference point for determining the impact of the WOCF initiative in later evaluative research. All interviews were conducted either face-to-face or by telephone, audio recorded, and transcribed into text documents [34]. The interview data was thematically analysed. Expectations prior to PEP, experiences of supervision, support during placement, supervision confidence, expectations of students, and student adaptation were themes that emerged from the data. Most interviews were with single respondents, although some had two and three participants. Forty-one respondents participated in these interviews (seven students, 25 supervisors/staff and nine WOCFs).

Further interviews were undertaken in 2018, with 19 participants (10 students, one supervisor, and eight WOCFs). The 2018 research used the same recruitment process as 2017, with emails sent to staff at host facilities. Students were also emailed and invited to participate in the research. This research was to gauge how the implementation process was being experienced and to highlight any problems with the model. Students represented the largest group of respondents during this period, and their feedback revealed areas where changes were required to improve PEP experiences.

In 2016, an information forum was held to disseminate information about the initiative and explain the implementation process. In addition to the 2017 and 2018 interviews, information forums and stakeholder workshops were also held with staff from host facilities. Stakeholder networking events were conducted to provide project updates, and receive feedback and ideas to further enhance the quality of student and supervisor experiences.

In 2018, an online survey was used to collect feedback from managers, supervisors, and other staff about their perspectives and experiences of students’ professional experience placements. A purposive sampling approach was used, with emails sent to participating healthcare facilities in WOCF regions. The sample population was 109, and 37 respondents completed two or more sections of the survey and were included in the data analysis. The survey collected both qualitative and quantitative data with a Likert 5-point scale of agreement/disagreement responses to statements about PEP. Some questions also invited free-text responses [34,35].

In 2020 WOCFs, managers, supervisors, and students were invited to participate in individual interviews. Recruitment was again undertaken by email and referral, with WOCFs encouraging participation in the research. Twelve respondents participated in the 2020 interviews. Unfortunately, this research commenced when the COVID-19 pandemic was declared, impacting progress of the research. Rather than undertaking face-to-face interviews, telephone interviews were preferred. The pandemic also curtailed participation rates as healthcare professionals were impacted with increased workloads and the research team made the decision to halt recruitment, recognising the pressures potential participants were enduring in their work environments.

On implementation of the initiative, registered nurses were appointed to WOCF roles to facilitate PEP within defined geographical regions across rural Tasmania. Each WOCF supports the healthcare facilities in their region with facilitation support they may not otherwise be able to provide themselves. In 2018, a lead WOCF was appointed following acknowledgement of the need for a principal that would support other WOCFs, and manage recruitment, training, and coordination of these roles across the state. The lead WOCF also connects WOCFs through hosting regular virtual professional development activities. This is an example of the responsiveness of the model. The need for a lead position was recognised through the feedback from other WOCFs, managers and supervisors.

WOCFs support supervisors and students during PEP. In the absence of students on placement, WOCFs work with staff in rural facilities to increase their capacity to host students by providing supervision training, resource development, and other activities identified as being beneficial to increasing the capability of staff to supervise students. Additional activities occur in response to the needs of facilities in each WOCF’s region and are not prescriptive. Consequently, facilities are supported in ways that address their unique needs, thereby ensuring they can provide students with positive placement experiences. Table A1 in Appendix A outlines the general position responsibilities of WOCFs.

## 3. Results

The results showed a progression of acceptance of the initiative within the rural PEP community from 2017 to 2020. The early data indicated some initial hesitancy about the project, to full acceptance and confidence in the WOCF position and the support WOCFs provide. For example:

*“I think there has been some difficulties in the WOCF understanding, or the support that the WOCF has received. Like any new position, there is going to be … a settling-in period. And I think probably that settling-in period has been to date, and hopefully now there will be some, you know, some more concrete, or some more—[WOCF] will feel that [WOCF] actually understands what it is [WOCF] is to do”.*

*(Supervisor Respondent 12—Director of Nursing)*

There was also early evidence of the responsiveness of WOCFs to the needs of individual facilities:

*“I found we were lacking in the orientation of the students to the facility, because everywhere is busy, and when the students arrive it is sort of, ‘great here you go—you’re buddied up with someone’—and then they hit the floor running. So what [WOCF] and myself have spoken about is giving the students a day or a 1/2 a day of orientation and we have worked on a package which is similar to what we give to our permanent staff … Together they get to meet everybody who’s doing the same placement as them and get to facilitate those sort of social relationships as well”.*

*(Supervisor Respondent 16, 2017—Nurse Unit Manager)*

By 2020, the data showed the WOCF role had wholly integrated into the milieu of PEP at host facilities:

*“Before the WOCF, there’s always challenges, we’ve had students that have been overly confident, under confident, language barriers, we’ve had issues where we haven’t known exactly what the students were supposed to be doing, yeah so we’ve had lots of little things … now I think that all that sort of stuff is a lot smoother … Yeah and if we did have any issues we would go to the WOCF, she’s sort of our go-to person … so now that we have got the WOCF everything is a much smoother process”.*

*(Supervisor Respondent 16, 2020—Nurse Unit Manager)*

There was also evidence of the increasing capacity of host facilities as a result of the initiative:

*“We usually just take one student at a time, and that’s just because we’re a small practice. We’re looking to grow, which is also why we take students, because it’s a good recruitment opportunity. Hasn’t worked yet, [laughs], but, you know, it’s one of our recruitment methods … last year we took two students … We usually take one at a time, and we took two last year and this year we’re having three”.*

*(Supervisor Respondent 27, 2020)*

Data from 2017 interviews indicated managers and supervisors were unsure about how the initiative would benefit their organisations. It also revealed many had previous experiences of PEP that were not always positive. The research also indicated WOCFs were unsure of their roles as they adjusted to their new responsibilities. Many WOCFs reportedly struggled to balance the responsibilities of the role with their clinical duties, particularly when their facilities were short-staffed. By 2020, the research data showed the WOCF model had become an embedded feature of PEP. Managers and supervisors demonstrated strong commitment to the initiative, which was expressed in statements referring to WOCFs as “our WOCF” and “our go-to person”. Importantly, the research indicated that host organisations were reliant on this support to provide positive placement experiences.

Importantly, WOCFs are critical to the effective delivery of placements for many host organisations. Now embedded to support PEP experiences, WOCFs monitor student learning and overall placement experiences and are alert to potential issues that could develop. When issues arise, their role includes addressing these matters and ensuring fair processes are enacted. WOCFs advocate on behalf of students as necessary, and skilfully address sensitive matters. This role is important to minimise potential negative impact on student placement experiences.

In summary, WOCFs support supervisors, are attuned to workplace cultures, and are mindful of matters that can adversely impact placement experiences. They ensure students have adequate information about host organisations and communities prior to placements. WOCFs nurture respectful relationships with, and between, supervisors and students. They facilitate learning opportunities, ensure students and supervisors have access to necessary resources, and provide consistency for host organisations, supervisors, and students. Hence, WOCFs are invaluable to positive placement experiences in rural Tasmania. Importantly, this may lead to students seeking employment opportunities within rural healthcare environments following successful completion of their course, as noted by Sutton et al. [2,25].

## 4. Discussion

The research feedback demonstrated that as WOCFs embraced their roles and gained confidence with how they support rural PEP in their regions, stakeholder confidence grew. The research feedback from WOCFs indicates they spend much of their time building strong relationships with host organisations to ensure placements are effective. This relationship-building comprises providing supervision training, information, and communication between the College and host organisations. Strong relationships with host organisations assist WOCFs to build the capacity of these organisations to facilitate increased PEP opportunities. Building capacity at host organisations necessarily includes building the capability of individual supervisors, which, in turn, increases supervisor confidence, develops workplace understanding of learner needs and capabilities (learning “atmospheres”), and provides alternative professional development pathways for staff. WOCFs also develop supportive relationships with students and advocate on their behalf, when necessary, to ensure fair processes are activated. The relationship-building capacity of WOCFs underpins the success of the initiative.

Although WOCFs provide considerable support to students during placements, it is their work with host organisations that has been crucial to improving the overall quality of placement experiences and has increased the capacity of these organisations. Staff within host organisations have a pivotal role in creating positive and meaningful student placement experiences [36] by creating authentic learning experiences, role modelling professional behaviours, and providing guidance and feedback to students to enhance their knowledge and skills [37]. To provide quality support to students, supervisors at host organisations require knowledge, skills, and resources related to facilitating effective student learning [38]. Workplace demands and student support requirements mean it is unlikely many supervisors will have undertaken supervisor training [39]. WOCFs are an appropriate resource to provide this support, including professional development regarding students’ assessment requirements and additional tailored information to facilitate student learning. Therefore, the WOCF initiative has the potential to provide transformative support before, during, and after PEP to host organisations, supervisors, and students.

A critical learning from the project is that although the primary objective of the initiative was to improve students’ experiences during PEP and promote rural workplaces as potential employment destinations, the evolution of the model revealed this is best achieved by supporting supervisors and staff at host facilities. Hence, the project inverses the object of the intervention in that students receive the most benefit when conferred with supervisors and staff at host facilities. That is, early in the project it was realised that, irrespective of the support students received from WOCFs during PEP, if the supervision they received at the host facility was less than optimal, then the quality of their experience was affected. Therefore, this project is novel because it directs support to supervisors who then raise the quality of their supervision of students. In this respect, the support to students is often indirect—which is a critical outcome of the project. Nonetheless, WOCFs also provide direct support to students where necessary.

## 5. Conclusions

From humble beginnings as a trial program in 2012, the WOCF initiative has matured into a central and vital component of quality placement experiences in rural Tasmania. From the trial of an innovative model, the initiative has developed, evolved, expanded, and matured to become an integral role within the nursing program at the University of Tasmania. Importantly, it is embedded as part of the milieu of PEPs with WOCFs providing crucial support to facilities, supervisors, and students to increase student placement capacity and improve placement experiences for all.

Positive student rural PEP can be an influential mechanism for addressing workforce shortages in rural health facilities. The necessary components for positive student placements are supervisors who can guide students, expose them to diverse experiences, initiate learning and teaching activities, and provide timely and appropriate feedback. As rural practice environments differ substantially to metropolitan settings, responsive support models are necessary to facilitate effective placements where host organisations, supervisors, and students are supported to ensure quality placements are experienced by all concerned. This requires a model of support that accommodates the specific needs of workplaces that often have limited resources. The WOCF model provides supervisors with quality resources to support best practices and enhances quality supervision. The WOCF model is a useful exemplar of structured support for PEP that enhances quality. It is responsive and adaptive to the environments of rural healthcare practices and is beneficial for improving quality and fostering positive and effective student experiences.

The research has shown how long-term commitment to an initiative is necessary to positively impact rural healthcare facilities and provide beneficial support to organisations providing PEPs to students as they develop into emergent healthcare professionals. Because of this initiative, students are exposed to healthcare provision in rural communities whilst remaining connected to the College and are guided and supported by WOCFs who are instrumental in developing the future rural healthcare workforce. Although it was students as future healthcare professionals who were the prime focus of the initiative, it was through the relationships developed between WOCFs and healthcare facilities that the overall objectives were realised. The next step for the project is longitudinal research tracking graduate outcomes and career destinations to determine the positive influence of the initiate on the rural healthcare workforce in Tasmania.

## Data Availability

The data are not publicly available due to the potential to identify individuals or groups within small rural communities.

## Appendix A

WOCF regions in Tasmania vary considerably—geographically, spatially, and demographically, and WOCFs need to be responsive and adaptive to the needs of their individual regions. The practical supports WOCFs provide vary between regions; however, particular aspects of the role remain consistent. Table A1 outlines the range of activities undertaken by all WOCFs from pre- to post-placement.

**Table A1:** General activities of WOCFs.

Activity Periods	Types of Activities
Pre-placement	Manage the pre-placement period by advising hosts ahead of time about students allocated to their facilities, including important student information. Contact students and give them information about the WOCF role, their contact information, and information about the host facility.
During placement	Orient students on arrival at host facilities. Complete student assessments during PEP. Debrief students throughout PEP. Act as advocate and/or confidant for students, as needed. Are the “go-to” person for students during placements. Value-add to learning experiences—for both students and supervisors. Oversee student learning. Manage problems with students in a timely way. Support students to be involved in their host communities. Work with managers and supervisors to ensure placements are delivered effectively.
After placement	Are the conduit for communication between College and host organisations. Feedback to facilities and feed-forward to the College.
Between placement	Provide additional resources to host organisations and supervisors when needed. Build relationships within rural communities. Negotiate access and/or vouchers for students to visit local tourist attractions within regions whilst on PEP.
